# Field Trials of Wild Entomopathogenic Fungi and Commercial *Steinernema carpocapsae* on the Large Pine Weevil (*Hylobius abietis* [L.]) Including an Assessment of Non-Target Effects

**DOI:** 10.3390/insects15120967

**Published:** 2024-12-04

**Authors:** Luis M. Quinzo-Ortega, William T. Swaney, Roger Moore, Robbie Rae, Christopher D. Williams

**Affiliations:** 1 School of Biological and Environmental Sciences, Liverpool John Moores University, Byrom Street, Liverpool L3 3AF, UK; l.m.quinzoortega@2020.ljmu.ac.uk (L.M.Q.-O.); w.t.swaney@ljmu.ac.uk (W.T.S.); r.g.rae@ljmu.ac.uk (R.R.); 2 Entomology, Tree Health, Forest Research, Northern Research Station, Roslin, Midlothian EH25 9SY, UK; roger.moore@forestresearch.gov.uk

**Keywords:** large pine weevil, entomopathogenic nematodes, entomopathogenic fungi

## Abstract

The large pine weevil (*Hylobius abietis* L.) is a major pest in European and Asian coniferous forests, particularly in managed plantations where clear-felling practices create ideal conditions for its population growth. Traditional management practices involving synthetic insecticides have limited efficacy in terms of reducing pest populations and pose environmental risks. This study evaluated the effectiveness of a wild entomopathogenic fungus (EPF) and the commercial entomopathogenic nematode *Steinernema carpocapsae* (EPN) as biological control agents (BCAs) against *H. abietis* in clear-felled spruce plantations in Wales and Scotland. Field trials used a randomised block design with three treatments (EPN full dose, EPF full dose and a combination of EPF+EPN at half doses each) compared to a control. Emergence trapping and destructive sampling were employed to assess treatment efficacy. All treatments significantly reduced weevil emergence, with the mixed treatment showing the greatest impact. Destructive sampling revealed strong associations between treatment type and infection outcomes in *H. abietis*, with a small but significant relationship between weevil developmental stages and infection types. Importantly, the treatments had no significant impact on the total abundance or taxon richness of non-target invertebrates. These findings suggest that wild EPFs alone and combined with EPNs are effective and environmentally safe alternatives to synthetic insecticides for managing *H. abietis* populations in managed forests.

## Simple Summary

The major insect pest of European plantation forests is the large pine weevil. Traditionally, this pest has been controlled by applications of synthetic pesticides. However, because of human health and environmental concerns, there is a need to develop alternative sustainable management strategies. One such strategy is the use of biological control agents. We trialled the efficacy of wild-collected insect-killing fungi and commercial round worms (nematodes) alone and in combination against immature stages of the large pine weevil, which live under the bark of freshly cut stumps. All treatments, as assessed by monitoring emergence with specialised traps, significantly reduced the weevil populations compared to the untreated controls, and the destructive sampling of stumps revealed strong associations between treatment type and infection outcomes. We also monitored non-target insects emerging from treated and untreated stumps. There was no effect on abundance, taxon richness or community composition of the control agents, indicating that they encountered negligible negative environmental consequences.

## 1. Introduction

*Hylobius abietis* (L.) (large pine weevil) has a widespread natural distribution in Europe and Asia. This weevil is highly attracted to freshly cut conifer stumps that release volatiles, such as alpha-pinene, that indicate the availability of wood material for egg laying [1,2]. The availability of stumps of fallen trees within which to reproduce is low in natural woodlands and, under natural conditions, is a limiting factor for the increase in *H. abietis* populations. However, the opposite scenario is found in expansive coniferous exploitations [3]. The harvesting of large coniferous plantations by clear-felling provides hundreds, or even thousands, of stumps that have made *H. abietis* populations increase, affecting the regeneration of such forests [2,4].

*H. abietis* lifecycle ranges from 12 to 36 months depending on the temperature (the climate change-related increase in temperature shortens the cycle and favours dispersion), starting as eggs laid below ground by the stumps [5,6]. Hatched larvae feed in the cambial layer until pupation and emergence of callow adults that mainly feed on the bark of young conifer saplings (often leading to their death by girdling), exposed lower stems and the upper part of coniferous and broadleaved trees [3]. A solitary adult *H. abietis* lethally affects several saplings up to five years old, when young trees can withstand debarking damage. Consequently, adults in high number are a pest for forest re-stocking, as they can kill all the new saplings when no protective measures are undertaken [4,7]. In Europe, 3.4 million ha of forest are threatened, producing estimated losses of €140 million per annum; meanwhile, public and private forests within the U.K. suffered estimated losses of €4.6 million in 1998 [4,8,9]. 

The integrated control of *H. abietis* in U.K. and Ireland can incorporate the pre-application in nurseries of acetamiprid (amongst other synthetic chemicals used under derogation) to protect the seedlings prior to forest planting, followed by an additional insecticide top-up spraying on site [3,8,10]. There is concern due to the potential environmental impacts of these substances, as they are considered “highly hazardous chemicals” according to the Forest Stewardship Council (FSC) guidelines [8,9]. For example, cypermethrin presents high toxicity to freshwater and other non-target organisms [11,12,13]. Moreover, the use of synthetic insecticides has a repulsive rather than a lethal effect on *H. abietis* and does not help to reduce the local populations of the pest [14,15].Alternatives to the use of synthetic chemicals against sapling damage by *H. abietis* include changes in silviculture practices such as mounding, delayed restocking, altered silvicultural practices based on risk management systems integrated with GIS and the application on stumps of the decaying fungus *Phlebiopsis gigantea* (Fr.), which makes the stumps unsuitable for oviposition [8,16,17,18,19,20,21,22]. Another alternative is the use of mechanical barriers such as collar guards and the application of a protective coating [3,23,24,25]; physical barriers, under determined circumstances, in the U.K. and Ireland can partially replace synthetic chemicals as part of an integrated approach to protect saplings [10]. A promising approach is the use of biological control agents (BCAs) that are a less environmentally impactful option amongst the alternative treatments when applied as biopesticides in an inundative fashion to target and supress *H. abietis* infestations [26,27]. Entomopathogenic nematodes (EPNs) and entomopathogenic fungi (EPFs) are considered environmentally safe BCAs for plants, vertebrates and non-target insects, even though they have a broad host range, and have the advantage that they can be mass-produced and have limited persistence in the environment [6,28,29,30,31]. 

EPN free-living infective juveniles (IJs) are the host-seeking stage that, first, find and enter the insect host via spiracles, the mouth, the anus and/or the cuticle and then, colonise and kill the insect by releasing symbiotic bacteria (*Xenorhabdus* sp. by Steinernematidae, or *Photorhabdus* sp. by Heterorhabditidae) that turn the host tissue into a nutrient medium that favours the continuity of EPN lifecycle until the release of numerous new IJs [32,33]. *Steinernema carpocapsae* is recognised as an “ambush forager” that sits and waits for its insect host, sometimes nictating on the substrate. The IJ stage is non-feeding and resistant and the only life stage of an EPN that can survive outside an insect host. The EPFs used as biopesticides against *H. abietis* include species from the genera *Metarhizium* and *Beauveria*, which infect by contact with their mycelium and asexual conidiospores in every stage of target insects with piercing–sucking mouthparts [34,35,36]. Both EPNs and EPFs have been reportedly used as biopesticides to target developing larvae and pupae of *H. abietis* in field trials, applied in an aqueous suspension drenched around stumps [5,9,36,37,38,39]. For example, Kapranas et al. (2017) [7], using the EPN *Steinernema carpocapsae* (Weiser, 1955) at the recommended operational full dose of 3.5 × 10^6^ IJs/500 mL per stump on field trials obtained suppression of emergence below the threshold of 20 adults of *H. abietis* per stump. In other works, similar suppression was obtained when *S. carpocapsae* was applied in half doses together with half doses of the EPF *Beauveria caledonica* Bisset and Widden (1.75 × 10^6^ IJs + 5 × 10^8^ conidiospores/500 mL per stump), displaying additive effects of these BCAs [36]. Later, Mc Namara et al. (2018) [9] also found additive effects for various pairs of EPNs and EPFs that provided different levels of reduction in the emergence of adult *H. abietis* up to 93% with respect to the control.

The aim of this paper is to build on this line of work by studying in clear-felled woodlands the effects of new wild EPFs that provided good control of adult *H. abietis* in the laboratory, examining them alone and in combination with the commercial EPN *S. carpocapsae.* We are interested not only in the effectiveness but also in the safety of these BCAs in relation to non-target stump-dwelling insects.

## 2. Materials and Methods

### 2.1. Sites for the Field Trials

The field trials were conducted in two clear-felled spruce plantations managed by the Forestry Commission, which had been felled for 15 to 18 months. The presence of *H. abietis* larval stages was confirmed through destructive sampling of stumps during July 2021 for the first site trial and during May 2022 for the second field trial. The first site, used for the 2021 trials, was felled around February 2020 in Abergynolwyn, Gwynedd, Wales (grid reference: SH675054), and the second site, used for the 2022 trials, was felled between October 2020 and February 2021 in Clatteringshaws, Scotland (grid reference: NX545791). Both sites were treated with wild entomopathogenic fungi (EPFs). The Welsh site, on 23/07/2021, was treated with *Metarhizium* sp. 15G (RMK-2011b according to BLAST likelihood analysis), and the Scottish site with *Beauveria bassiana* isolate 35G (DAOM210087 according to BLAST likelihood analysis) on 16 June 2022. These wild fungi were obtained from nature reserves in Lancashire [40]. Additionally, both sites received treatments with the commercial entomopathogenic nematode (EPN) *Steinernema carpocapsae* (Nemasys^®^ C), supplied by BASF, Widnes, UK.

The treatments on each site were arranged in a randomised block design of twenty blocks (ten repetitions for emergence trapping and ten for destructive sampling). Each block replicate included three treatments (EPN full dose, EPF full dose and EPF + EPN half doses) and a control. 

### 2.2. Treatments and Application

The mass production of EPF conidiospores was achieved by plating the selected wild fungi in twenty sterile 9 cm Petri dishes containing potato dextrose agar (PDA) medium (Oxoid Ltd., Basingstoke, Hampshire, UK). The plates were incubated in dark conditions at 27 °C for two weeks prior to field application. The conidiospores were rinsed out of the plates the day before the application using sterile distilled water mixed with 0.03% (*v*/*v*) viscous Tween-80 (Sigma-Aldrich, Dorset, UK), and the content of four plates at a time was collected into 50 mL Falcon^TM^ tubes (Scientific Laboratory Supplies, Nottingham, UK) and kept at 4 °C overnight. The conidiospores were counted using a haemocytometer (Hawksley Ltd, Lancing, West Sussex, UK) placed under a light microscope (400× magnification), and their concentrations were obtained following Lacey (1997) [41]. 

At the start of the field application, Nemasys^®^ powder containing *S. carpocapsae* was suspended in 1 L measuring cylinders using distilled water. The active EPNs were then counted from five 200 μL aliquots using a binocular stereo microscope to estimate their concentrations per litre. The suspensions were finally diluted into 5 L containers to the desired concentrations and aerated with aquarium pumps [36]. 

The full-dose treatments included commercial nematodes at a rate of 3.5 × 10^6^ nematodes per 500 mL and wild entomopathogenic fungi at a rate of 10^9^ conidiospores per 500 mL. The half-dose treatment (HalfMix) combined 1.75 × 10^6^ nematodes with 5 × 10^8^ conidiospores per 500 mL. The control treatment consisted of distilled water. All the treatments were diluted in distilled water, and when the conidiospores were included, the aqueous treatments contained 0.03% (*v*/*v*) viscous Tween-80. The treatments were applied by drenching the top perimeter of each stump, above soil level, allowing the biological agents to penetrate between the bark and the stump effectively.

### 2.3. Assessment of Efficacy

The efficacy of the treatments was evaluated by destructive sampling and emergence trapping, complemented by a mark–recapture experiment associated with the latter. Three weeks after treatment application, 10 blocks where destructively sampled following a similar approach to that of Dillon et al. (2006) [5]. All the bark from the main stump bole and its roots up to 50 cm, was carefully removed using a chisel, while determining in situ the developmental stage of *H. abietis* (larva, pupa and adult) and the infection status (alive, nematode-killed, fungus-killed or indeterminate). For each *H. abietis*, the depth and distance from the bole of the stump were recorded. The collected weevils were placed in 24-well plates (Greiner Bio-One Ltd., Stonehouse, Gloucestershire, UK) using clean forceps and monitored in the laboratory for two weeks at room temperature (22 °C) to check for the presence of EPF mycelium or EPN. 

Modified emergence traps designed by Moore (2001) [1] and a parallel mark–recapture experiment were deployed four weeks after the application of the treatments on the remaining 10 blocks. For the mark–recapture test, three freshly cut Sitka spruce billets (approximately 30 cm long by 15 cm diameter) were stacked in order to attract adult *H. abietis* from the surrounding area and placed in the centre of each block on top of a PDA Petri dish (9 cm diameter) containing the sporulated wild *Metarhizium* sp. 17G (RMK-2011b according to BLAST likelihood analysis) used as a bioinsecticide [40]. The aim of the mark–recapture experiment was to see if those weevils escaping control in the treated stumps could be attracted to billets in the field and subsequently controlled with the EPF, which was placed under each billet. The traps and billets were checked every week until *H. abietis* emergence ceased in November. Adults of *H. abietis* from the control emergence traps were collected and taken to the laboratory, while the ones collected from the treatment traps were colour-marked on the right elytra with quick-dry nail polish (Sally Hansen, New York, NY, USA) for identification (if recaptured) and released back into the field. 

For each collection of weevils from the emergence traps in the second field trial (Clatteringshaws), non-target invertebrates that emerged were also collected. Initially, for all of these invertebrates, the Order or another higher classification level was determined. These taxa comprised Coleoptera, Diptera, Hymenoptera, Lepidoptera, Myriopoda, Arachnida, Opilionidae, Auchenorhyncha, and Collembola.

Following this initial classification, all Diptera were determined to the family level, and ground beetles (Carabidae) were determined to the species level to provide a second, more detailed, taxon list. The Diptera were identified using Unwin’s guide [42], and the Carabidae were identified using Luff’s guide [43]. Following the identification and tabulation of the non-targets, total abundance, broad taxon richness and narrow taxon richness were compared among the treatments, statistically. 

### 2.4. Statistical Analysis

The emergence data were analysed with R v.4.4.1 (R Core Team, Vienna, Austria) [44]; all other univariate analyses were performed on SPSS Statistics 29.0 (IBM Corp., Armonk, NY, USA), and all multivariate analyses were performed on PC-Ord v.7 (MjM Software Design, Gleneden Beach, OR, USA).

The emergence data from Wales (2021) and Scotland (2022) were analysed using a separate negative binomial generalised linear mixed model (GLMM) suitable for count data using the *lme4* R package [45], with treatment included as a fixed factor and replicate block as a random factor. Model fit was checked using the *performance* R package [46], and likelihood ratio tests (LRTs) were used to determine the effect of each treatment. The *emmeans* R package [47] was used to calculate the estimated marginal means, and Dunnett’s contrasts to compare each treatment group against the control and identify statistically significant reductions in emergence.

The chi² test was performed over destructively measured data from Scotland (2022) using cross-tabulation analysis to evaluate the relationships between the infection types across the different treatments, where standardised residuals (Z scores) were calculated to identify significant deviations from the expected frequencies by random distribution, with a threshold of ±1.96 indicating statistical significance. Additionally, the relationship between *H. abietis* developmental stage and infection type was analysed using the same chi² test and Z scores.

The Bliss independence model [45] was applied to destructive data to evaluate the interaction between EPF and EPN in the HalfMix treatment. The expected infection rate (*I_Bliss_*) was calculated using the formula *I_Bliss_* = *I_EPF_ + I_EPN_*− (*I_EPF_ × I_EPN_*), where *I_EPF_* is the infection rate in the half-dose EPF treatment, and *I_EPN_* is the infection rate in the half-dose EPN treatment. The chi-square value (Chi^2^_Bliss_) was calculated using the formula *Chi*^2^*_Bliss_ = (I_NF_ − I_Bliss_)*^2^*/I_Bliss_*, where *I_NF_* is the observed infection rate for the HalfMix treatment. The *p*-value for each Chi^2^_Bliss_ (with 1df) was determined using R (version 4.4.0) with the command >1-pchisq(Chi^2^_Bliss_,df). Sinergy (S) was calculated as *S* = *I_NF_ − I_Bliss_*, where *S* > 0 indicates a synergistic effect, *S* = 0 indicates an additive effect, and *S* < 0 indicates an antagonistic effect between the agents. 

The impact of the treatments on non-target invertebrates was assessed with one-way ANOVA. The raw data for the broad and narrow taxa are shown in Table S1 and Table S2, respectively. To further investigate compositional differences among the treatments, two ordinations (PCoA) were performed: one on the broad taxa, and one on the narrow taxa. Additionally, to assess the effect of the grouping variables (treatment and block) on the composition, the multi-response permutation procedure (MRPP) was performed again on the broad taxa and narrow taxa, separately. 

## 3. Results

### 3.1. Emergence

**Figure 1:**
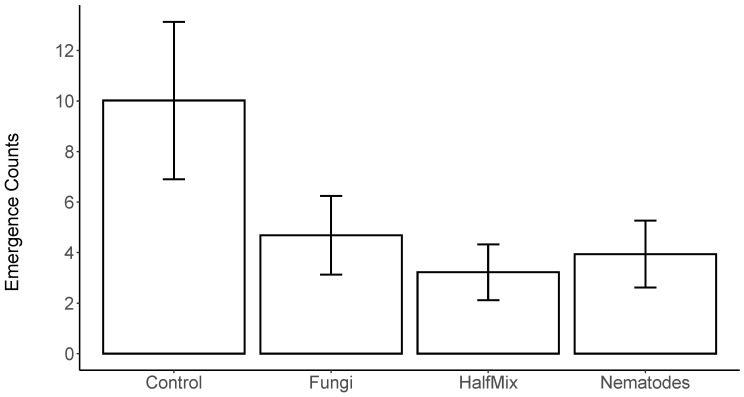
Emergence counts of *H. abietis* in Scotland from July to October 2022 exposed to full doses of EPN (nematode) and EPF (fungus); half-doses of combined EPF + EPN (HalfMix) and control. Counts are estimated marginal means from a negative binomial generalised linear mixed model, with treatment as a fixed factor, and replicate block as a random factor; error bars represent ± SE.

The GLMM analysis found a significant effect of the treatment on the data for weevil emergence in Scotland in 2022 (LRT Chi^2^ = 12.274, df = 3, *p* = 0.007), and Dunnett’s contrasts showed significant reductions in emergence for all treatments compared to the control (for all, *p* < 0.05, Figure 1), with the most pronounced reduction in relative emergence observed for the HalfMix treatment (Table 1). However, there was no statistically significant overall effect of the treatments on weevil emergence in the GLMM analysis of the 2021 data from Wales (LRT Chi^2^ = 5.544, df = 3, *p* = 0.136). 

### 3.2. Destructive Sampling 

The chi-square test revealed a highly significant association between treatment and infection type targeting developing and adult *H. abietis*, indicating a real difference between those categories (Chi^2^ = 186.23, *p* < 0.001) (Table 2). The EPF treatment significantly increased the number of fungus-infected *H. abietis* (Z = 7.6), while the EPN and HalfMix treatments increased the number of nematode-infected weevils (Z = 4.7 and Z = 3.7, respectively). The proportion of alive (non-infected) *H. abietis* in the control treatment was statistically higher than expected by random infection (Z = 4.4), while the non-infected proportions in the HalfMix and nematode treatments were significantly lower than expected (Z = −2.4 and Z = −2.4, respectively). The relationship between developmental stage of *H. abietis* and infection type was also significant, suggesting that different infection types affect each developmental stage in the field (Chi^2^ = 13.84, *p* = 0.031) (Table 2).

The population structure of the weevils in the Scottish field trial is shown in Table 3. As can be seen, the majority of the targeted individuals were larvae, followed by pupae and then a small proportion of callow adults.

In terms of effect size, according to Cohen (1988) [48], the relationship between infection type and treatment type was medium and statistically significant (Cramer’s V = 0.394, *p* < 0.001). Additionally, a small but statistically significant relationship was found between *H. abietis* developmental stage and type of infection (Cramer’s V = 0.132 with *p* = 0.031).

The analysis of the interaction between EPF and EPN revealed that the total observed infection rate (*I_NF_* = 0.5463) exceeded the Bliss model prediction (*I_Bliss_* = 0.3790), indicating a synergistic interaction between EPN and EPF in the HalfMix treatment. However, the low chi^2^ value (Chi^2^_Bliss_ = 0.0739) and the high *p*-value (*p*-value = 0.7858) suggested that the deviation was not statistically significant. A block-level interaction analysis found predominantly synergistic effects, with some antagonistic effects, although none was statistically significant (Table 4).

Nematode- and fungus-infected *H. abietis* organisms were found at different depths and distances from the stump bole. *S. carpocapsae* actively infected at depths of 36 cm underground and spread longitudinally along superficial stump roots up to 49 cm. In the case of *H. abietis* infected by *B. bassiana* 35G, the maximum infection depth was 23 cm underground and 19 cm longitudinally (Table 5).

### 3.3. Mark–Recapture

The mark–recapture experiment results were limited due to the low numbers of recaptures, which meant that neither blocks nor treatments could be statistically tested for differences in recaptures. Billets attracted adult *H. abietis*, and it is notable that the field trial in Scotland attracted more weevils overall than did the billets in Wales. Only a very small number of insects were adults released from the emergence traps (Table 6). Death could not be confirmed, as this experiment was intended to allow the weevils to freely feed and move to enter in contact with the conidiospores placed under the billets. For both field trials, no weevil adults were observed to have died as the result of EPF infection.

### 3.4. Effects of the Treatments on Non-Targets

**Figure 2:**
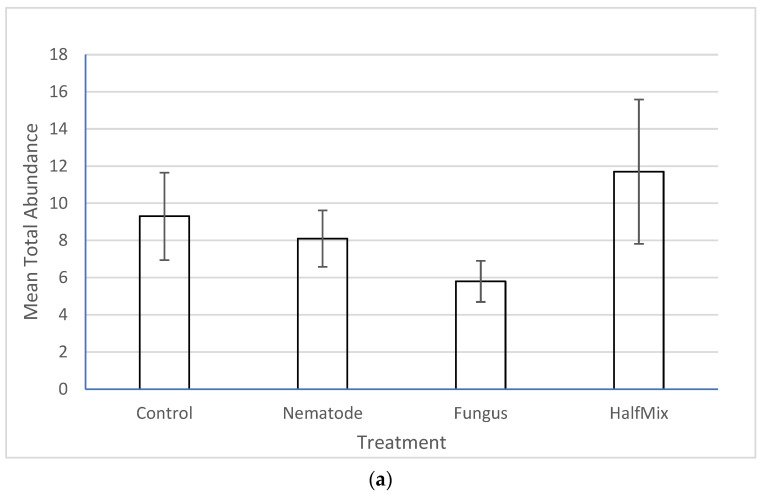

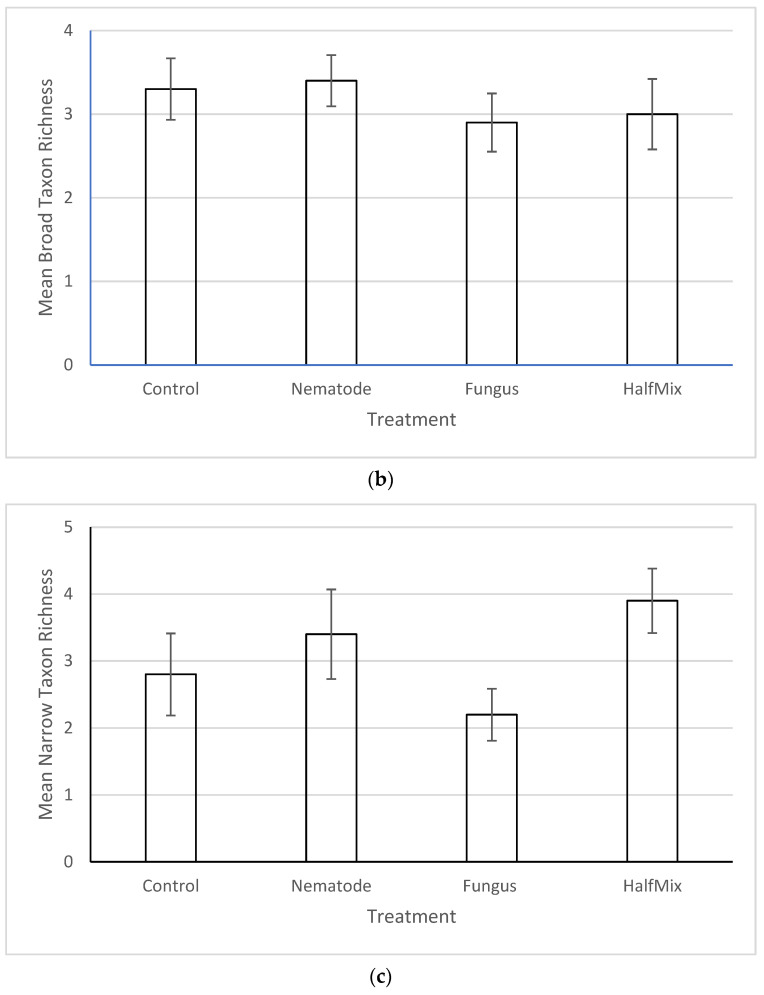
Effects of the treatment (nematode, fungus, mixed and control) on (**a**) non-target total abundance, (**b**) broad taxon richness and (**c**) narrow taxon richness. None of the differences are significant (one-way ANOVA, *p* > 0.05 in all cases). Error bars are +/− SE. For definitions of broad and narrow taxa, see Section 2.

There was no significant effect of the treatments on the total abundance (Figure 2a), broad taxon richness (Figure 2b, Supplementary Table S1) or narrow taxon richness (Figure 2c, Supplementary Table S2) of non-targets. In all cases, one way ANOVA returned *p* values greater than 0.05.

**Figure 3:**
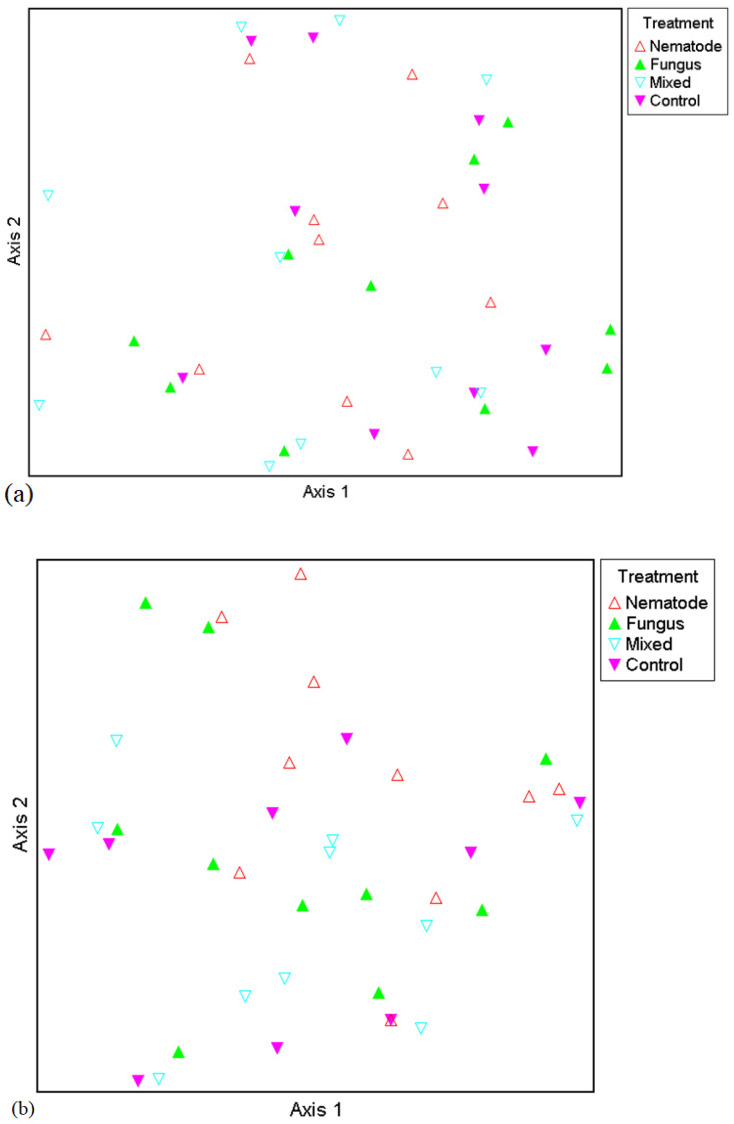
Principal co-ordinates analysis (PCoA) for broad taxa (**a**) and narrow taxa (**b**). As can be seen in both plots, there is no clustering of traps with respect to treatment. This was more formally tested with the multi-response permutation procedure (MRPP)—see Table 6. For definitions of broad and narrow taxa, see Section 2.

Two ordinations are presented: firstly, for the broad taxa, and secondly for the narrow taxa (Figure 3a and Figure 3b, respectively). These are multidimensional scaling plots (also called principal co-ordinates analysis [PCoA]). As can be seen in Figure 3a,b, there is no clustering of traps with respect to the treatment from which they were drawn. This indicates that compositional effects of treatments were not evident in the dataset. This was further borne out by the results of the multi-response permutation procedure. In all cases, both treatment and block effects were low (see Chance-Corrected Within-Group Agreements [*A*], which were low for all metrics). The *p* values simulated using Monte Carlo randomisation never reached significance for either the broad taxa or the narrow taxa with respect to treatment effects or block effects (Table 7).

## 4. Discussion

The results from the present field studies provide compelling evidence for the effectiveness of the wild EPF *B. bassiana* 35G alone and together with the commercial EPN *Steinernema carpocapsae* as BCAs against *H. abietis* in clear-felled spruce plantations in Scotland. These findings contribute to the growing body of literature advocating for sustainable and environmentally benign pest management strategies in forestry [27].

The data clearly indicate a statistically significant reduction in weevil emergence caused by all treatment regimens (EPFs alone, EPNs alone or a combination of both) compared to the control. Notably, the application of *B. bassiana* 35G at a full dose demonstrated the most substantial impact on weevil emergence (using corrected data excluding an outlier), thereby affirming the potential of this wild EPF as a potent standalone treatment. This killing potential against adult *H. abietis* was also previously demonstrated under controlled laboratory conditions at different concentrations [40]. The combination treatment (HalfMix) and the full dose EPN treatment also showed considerable efficacy, albeit to a slightly lesser extent than the full EPF treatment. This suggests that mixed doses could provide adequate control, as documented in previous studies [9,36]. Interaction analysis using the Bliss independence model indicated overall synergistic effects in the HalfMix treatment between half doses of B. *bassiana* 35G and *S. carpocapsae*. However, these effects were not statistically significant, and block-level analyses revealed some variability, including occasional antagonistic interactions. Such variability highlights the potential influence of environmental conditions and experimental consistency on the interaction dynamics of EPFs and EPNs against *H. abietis*. Nonetheless, the overall results support the additive nature of combined treatments, demonstrating their potential to enhance pest control efficacy while reducing the required dosages. 

The significant reduction in weevil emergence in the Scottish trial underscores the adaptability and potential success of these biological agents under varying environmental conditions. This finding is particularly relevant considering the ongoing challenges posed by climate change, which is likely to alter the lifecycle and dispersal patterns of *H. abietis* and other forest pests [2]. The observed efficacy across different sites further bolsters the argument for a wider adoption of these BCAs in forest management practices across the U.K. and potentially in other regions facing similar pest challenges [27].

It may be suggested that the higher number of adult *H. abietis* attracted to billets in the field trial in Scotland compared to the trial in Wales may be a result of the closer proximity in Scotland of mature undisturbed conifer plantations, which feasibly supply a spillover of adult weevils. This highlights a danger that attract-and-kill approaches could lure in more weevils and make matters worse. Alternatively, it could simply be that weevil populations are larger in Scotland. The low numbers of recaptures from treatment-released stumps highlights the limitation of relying on billets as attractants for adult weevils. The development of lures including alpha-pinene may provide more effective alternatives in lure-and-kill approaches for the management of adult *H. abietis*.

A critical aspect of deploying BCAs in the field is ensuring that they do not adversely affect non-target organisms [49]. Previous work [6] has shown that whereas site and tree species (of the stump) have a significant effect on the species richness, abundance and community composition of non-target Coleoptera, nematode treatment had no effect on these variables. Our results, from a more limited dataset, support these conclusions for invertebrates in general and further show that also entomopathogenic fungi have no effect on these variables. The results of the present study are particularly encouraging in this regard. The statistical analyses revealed no significant impact of any of the treatments on the abundance or taxon richness of non-target invertebrates. The ordination analyses and subsequent MRPP tests further corroborated the absence of compositional changes in the invertebrate community structure, reinforcing the conclusion that wild EPFs and commercial EPNs can be integrated into pest management strategies without detrimental effects on biodiversity. These findings are of paramount importance as they support the safety profile of these BCAs, thereby addressing a major concern associated with the use of synthetic insecticides, which are often non-selective and can cause collateral damage to beneficial organisms within the ecosystem [11,12,13].

The relationship between infection type (fungal or nematode) and the developmental stage of *H. abietis* indicated a statistically significant association in infecting and reducing the population of *H. abietis* across different developmental stages. This is important as, to date, the wild EPF *B. bassiana* 35G had only been shown to infect adult *H. abietis*, while in this study, we proved it efficient against pupae and larvae. The differential infection rates observed suggest that the timing and method of application could be critical in maximising the impact of these BCAs. For instance, studying how this wild EPF targets the different developmental stages of *H. abietis* could help to target those stages most susceptible to infection and thereby enhance the overall treatment efficacy and reduce the likelihood of pest resurgence.

These findings have important implications for the future of integrated pest management (IPM) in forestry. The demonstrated effectiveness of wild EPFs and EPNs suggests them as a viable alternative to synthetic insecticides, aligning with broader environmental goals to reduce chemical inputs in agriculture and forestry. The use of BCAs could be particularly beneficial in managed forest ecosystems, where the risk of pest outbreaks is exacerbated by practices such as clear-felling.

However, while the results are promising, further research is warranted to optimise the application protocols for these or new BCAs. This includes investigating the optimal timing, dosage and combination of EPFs and EPNs to ensure maximum efficacy across different forest types and climatic conditions. Additionally, long-term studies are needed to assess the persistence of these agents in the environment and their potential cumulative effects on both target and non-target organisms over time.

## 5. Conclusions

In conclusion, the data from this study provide strong support for the use of EPFs and EPNs as effective and environmentally safe tools in the management of *H. abietis* populations. Their integration into IPM strategies could significantly enhance the sustainability of forest management practices, reducing reliance on synthetic insecticides and mitigating their associated environmental risks. This research contributes to the growing evidence base that supports a paradigm shift towards more ecologically sound pest management solutions in forestry.

## Figures and Tables

**Table 1:** Scotland (2022) adult *H. abietis* emergence negative binomial generalised linear mixed model analysis from a randomised block design with 10 repetitions per treatment, with pairwise Dunnett’s contrasts between treatments and control. (*) Statistically significant values < 0.05.

LRT		Dunnett’s Contrasts (vs. Control)			
Chi^2^	*p*-Value	Contrast	Relative Emergence Ratio	Std. Error	Sig.
12.274	0.007	Fungi vs. Control	0.468	0.149	0.046 *
		Nematodes vs. Control	0.322	0.107	0.002 *
		HalfMix vs. Control	0.393	0.130	0.013 *

**Table 2:** Scotland (2022) *H. abietis* destructive sampling cross-tabulation chi^2^ analysis from a randomised block design with 10 repetitions per treatment. Indeterminate means Indeterminate cause of death. (*) indicates *p*-value < 0.05.

		Infection					
		Alive	Nematodes	Fungi	Indeterminate.	Chi^2^	Cramer
Treatment	**control**	113	0	2	2		
	% within treatment	96.60%	0.00%	1.70%	1.70%		
	% within infection	44.30%	0.00%	10.00%	18.20%		
	Z test (1.96)	4.4	−5.8	−1.6	−0.7		
	**fungi**	37	0	15	1	Value	Value
	% within treatment	69.80%	0.00%	28.30%	1.90%	186.23	0.3940
	% within infection	14.50%	0.00%	75.00%	9.10%		
	Z test (1.96)	0.50	−3.90	7.60	−0.40		
	**halfMix**	49	51	2	6	***p*-value**	***p*-value**
	% within treatment	45.40%	47.20%	1.90%	5.60%	<0.001 *	<0.001 *
	% within infection	19.20%	45.10%	10.00%	54.50%		
	Z test (1.96)	−2.40	3.70	−1.50	1.80		
	**nematodes**	56	62	1	2		
	% within treatment	46.30%	51.20%	0.80%	1.70%		
	% within infection	22.00%	54.90%	5.00%	18.20%		
	Z test (1.96)	−2.40	4.70	−2.10	−0.70		
Developmental stage	**adults**	26	4	1	1		
	% within development	81.30%	12.50%	3.10%	3.10%		
	% within infection	10.20%	3.50%	5.00%	9.10%		
	Z test (1.96)	1.20	−1.70	−0.50	0.10	Value	Value
	**larvae**	142	79	13	10	13.84	0.1320
	% within development	58.20%	32.40%	5.30%	4.10%		
	% within infection	55.70%	69.90%	65.00%	90.90%		
	Z test (1.96)	−1.10	1.20	0.20	1.30	***p*-value**	***p*-value**
	**pupae**	87	30	6	0	0.031 *	0.031 *
	% within development	70.70%	24.40%	4.90%	0.00%		
	% within infection	34.10%	26.50%	30.00%	0.00%		
	Z test (1.96)	0.90	−0.80	−0.10	−1.80		

**Table 3:** *Hylobius abietis* population structure in Scotland (2022) from the destructive sampling data cross-tabulation analysis of 10 blocks.

Population Structure		
	Total	%
Adults	32	8.0%
Larvae	244	61.2%
Pupae	123	30.8%

**Table 4:** Scotland (2022) *H. abietis* destructive sampling Bliss independence model analysed by each of the 10 blocks and in total.

	*I_NF_*	*I_Bliss_*	Chi^2^_Bliss_	*p*-Value	Synergy	
Total	0.546296296	0.37899579	0.073851637	0.785809	0.167301	Synergy
Block5	0.285714286	0.5	0.091836735	0.761855	−0.21429	Antagonist
Block9	0.764705882	0.6125	0.03782307	0.845799	0.152206	Synergy
Block10	1	0.5	0.5	0.4795	0.5	Synergy
Block11	0.8	0.409090909	0.373535354	0.541084	0.390909	Synergy
Block12	0	0.352941176	0.352941176	0.552453	−0.35294	Antagonist
Block13	0.285714286	0.428571429	0.047619048	0.827259	−0.14286	Antagonist
Block16	0.647058824	0.340697674	0.275485161	0.599676	0.306361	Synergy
Block18	0.296296296	0.25	0.49	0.483927	0.35	Synergy
Block19	0.5	0.25	0.25	0.617075	0.25	Synergy
Block20	0.9	0.3125	1.1045	0.293281	0.5875	Synergy

**Table 5:** Distances of types of infection on *H. abietis* population in Scotland (2022) from destructive sampling data of 10 blocks.

Infection				
	Alive	Nematodes	Fungi	Indeterminate
Depth (cm)				
Mean	−16.359	−15.681	−9.4	−13.364
Median	−17	−15	−9	−15
Minimum	−40	−36	−23	−21
Maximum	23	−3	2	−1
**cm to Bole**				
Mean	5.86	5.03	2.15	3
Median	0	0	0	0
Minimum	0	0	0	0
Maximum	34	49	19	15

**Table 6:** Total mark–recapture of adult *H. abietis* by treatment and by block in Scotland (2022).

	Wales 2021			Scotland 2022		
	Marked–Released	Total on Billets	Recaptured	Marked–Released	Total on Billets	Recaptured
Block1	1	3	0	6	47	0
Block2	2	1	0	8	35	0
Block3	20	13	5 Fungi	13	38	0
Block4	0	1	0	6	27	2 Fungi
Block5	0	5	0	20	38	1 Nematode
Block6	0	3	0	25	56	0
Block7	3	3	0	0	36	0
Block8	2	3	0	34	27	1 Fungi
Block9	3	6	0	16	18	0
Block10	1	0	0	6	18	0

**Table 7:** Results of the multi-response permutation procedure (MRPP) on broad taxa and narrow taxa with respect to two different grouping variables: treatment and block. Chance-Corrected Within-Group Agreement (*A*) is a measure of within-group homogeneity, and the simulated *p* value (Monte Carlo simulation) was non-significant for all grouping combinations.

Dataset	Grouping Variable	Chance-Corrected Within-Group Agreement (*A*)	*p*-Value
Broad Taxa	Treatment	−0.011	0.653
Broad Taxa	Block	−0.024	0.694
Narrow Taxa	Treatment	−0.011	0.860
Narrow Taxa	Block	0.012	0.261

## Data Availability

All data are available from the corresponding author (C.D.W.) upon reasonable request.

## Supplementary Materials

The following supporting information can be downloaded at https://www.mdpi.com/article/10.3390/insects15120967/s1, Table S1: Means and standard deviations obtained by Log(x + 1) and non-transformed data for Scotland 2022. Table S2: Broad taxa composition (Non-targets) at each trap for the Scottish Field Trial. Table S3: Narrow taxa composition (Non-targets) at each trap for the Scottish Field Trial 2022.
