# Supplementary material for: Field Trials of Wild Entomopathogenic Fungi and Commercial Steinernema carpocapsae on the Large Pine Weevil (Hylobius abietis [L.]) Including an Assessment of Non-Target Effects
Source: Insects. 2024 Dec 4;15(12):967. doi: 10.3390/insects15120967 (PMC11677840; doi:10.3390/insects15120967)
Supplement: Supplementary file 1 [file insects-15-00967-s001.zip › insects-3251108-supplementary.pdf]

Supplementary material

Table S1: Means and standard deviations obtained by Log(x + 1) and non-transformed data for Scotland 2022.

**Minus outlier**	**Mean**	**Std. deviation**	**All data**	**Mean**	**Std. deviation**
Fungi	4.33	1.886	Fungi	7	3.155
Nematodes	4.6	1.431	Nematodes	4.6	1.431
HalfMix	3.8	0.757	HalfMix	3.8	0.757
Control	12.9	3.15	Control	12.9	3.15
LogFungi	0.548	0.13385	LogFungi	0.6437	0.15328
LogNematodes	0.6358	0.10508	LogNematodes	0.6358	0.10508
LogHalfmix	0.6236	0.07803	LogHalfmix	0.6236	0.07803
LogControl	1.0107	0.12127	LogControl	1.0107	0.12127

Table S2: Broad taxa composition (Non-targets) at each trap for the Scottish Field Trial.

	Coleoptera	Hymenoptera	Diptera	Myriapoda	Lepidoptera	Arachnida	Auchenorrhyncha	Opilionidae	Collembola	Total abundance
1 Nematode	3	0	1	0	0	0	0	0	0	4
1 Fungi	1	0	2	0	0	0	1	0	0	4
1 Mixed	0	0	6	0	0	1	0	3	0	10
1 Control	1	3	1	0	0	1	0	0	0	6
2 Nematode	5	3	4	0	0	0	0	0	0	12
2 Fungi	2	0	0	0	0	0	1	0	0	3
2 Mixed	31	2	1	0	0	1	0	3	0	38
2 Control	1	1	0	0	0	0	0	0	0	2
3 Nematode	7	0	1	0	0	0	0	2	0	10
3 Fungi	2	1	1	0	0	0	0	0	0	4
3 Mixed	1	4	2	0	0	0	0	0	0	7
3 Control	2	1	0	0	0	1	0	0	0	4
4 Nematode	1	1	4	0	0	0	0	1	0	7
4 Fungi	0	0	0	0	0	0	1	0	0	1
4 Mixed	0	0	3	0	0	0	0	0	0	3
4 Control	5	0	1	0	0	0	0	0	0	6
5 Nematode	4	0	1	0	0	1	1	0	1	8
5 Fungi	6	2	0	0	0	0	0	1	0	9
5 Mixed	3	2	5	0	1	0	0	0	1	12
5 Control	12	1	2	0	0	2	0	6	0	23
6 Nematode	4	1	3	0	0	0	0	1	0	9
6 Fungi	2	1	0	0	0	0	0	0	0	3
6 Mixed	20	0	6	0	0	1	0	0	3	30
6 Control	2	3	1	0	0	0	0	0	0	6
7 Nematode	1	0	1	0	0	0	0	1	0	3
7 Fungi	0	1	2	0	0	1	0	1	0	5
7 Mixed	4	0	0	0	0	0	0	3	0	7
7 Control	12	1	7	0	0	0	0	2	1	23
8 Nematode	1	2	1	1	0	0	0	0	0	5
8 Fungi	0	3	1	0	0	1	1	6	0	12

8 Mixed	2	0	1	0	0	1	0	0	0	4
8 Control	1	1	5	0	0	1	0	0	0	8
9 Nematode	11	0	5	0	0	1	0	0	2	19
9 Fungi	7	1	1	0	0	0	0	0	0	9
9 Mixed	2	0	1	0	0	0	0	0	0	3
9 Control	3	0	0	0	0	0	0	4	0	7
10 Nematode	0	0	3	0	0	0	0	1	0	4
10 Fungi	1	0	6	0	0	1	0	0	0	8
10 Mixed	1	0	2	0	0	0	0	0	0	3
10 Control	4	0	3	0	0	0	0	1	0	8

Table S3: Narrow taxa composition (Non-targets) at each trap for the Scottish Field Trial 2022.

	Stratiomyidae	Rhagionidae	Mycetophilidae	Periscelididae	Syrphidea	Ceratopogonidae	Lauxaniidae	Dolichopodidae	Bibionidae	Tipulidae	Tachinidae	Patrobus atrofus	Bradycellus harpalinus	Notiophilus biguttatus	Pterostichus niger	Carabus problematicus	Nebria brevicollis	Trechus quadristriatus	Trichoceridae	Pterostichus madidus	Carabus glabratus	Total abundance
1 Nematode	0	0	0	0	0	0	0	0	0	0	0	0	0	0	0	0	0	0	1	3	0	4
1 Fungi	0	0	0	0	0	0	0	0	1	1	0	0	0	0	1	0	0	0	0	0	0	3
1 Mixed	0	0	1	0	1	2	0	0	0	1	1	0	0	0	0	0	0	0	0	0	0	6
1 Control	0	0	0	0	1	0	0	0	0	0	0	0	0	0	0	1	0	0	0	0	0	2
2 Nematode	0	1	0	0	0	3	0	0	0	0	0	0	0	0	1	0	1	0	0	3	0	9
2 Fungi	0	0	0	0	0	0	0	0	0	0	0	0	0	0	0	2	0	0	0	0	0	2
2 Mixed	0	0	1	0	0	0	0	0	0	0	0	0	0	0	2	1	0	0	0	0	0	4
2 Control	0	0	0	0	0	0	0	0	0	0	0	0	0	0	0	0	0	0	0	0	0	0
3 Nematode	0	0	0	0	0	0	0	0	0	0	0	0	0	0	1	0	0	0	1	0	0	2
3 Fungi	0	0	0	0	1	0	0	0	0	0	0	0	0	0	0	0	0	0	0	1	0	2
3 Mixed	0	0	0	0	1	0	0	0	0	1	0	0	0	0	2	0	0	0	1	1	0	6
3 Control	1	0	0	0	0	2	0	0	0	0	0	0	0	0	0	0	0	0	0	0	0	3
4 Nematode	0	0	1	1	0	1	0	0	0	0	0	0	0	0	1	1	0	0	0	1	0	6
4 Fungi	0	0	0	0	0	0	0	0	0	0	0	1	0	0	0	0	0	0	0	0	0	1
4 Mixed	0	1	0	0	0	1	0	0	0	0	0	0	0	0	0	0	0	0	1	0	0	3
4 Control	0	0	0	0	0	0	0	0	0	1	0	0	0	0	0	0	0	0	0	0	0	1
5 Nematode	1	0	0	0	0	0	1	0	0	0	0	0	0	0	0	0	0	0	0	2	0	4
5 Fungi	0	0	0	0	0	0	0	0	0	0	0	0	0	0	0	0	0	0	0	3	0	3
5 Mixed	0	2	0	0	0	1	0	0	0	3	1	0	0	0	0	0	1	0	0	0	0	8
5 Control	0	1	0	0	0	0	0	0	1	0	0	0	0	9	0	0	0	0	0	1	0	12
6 Nematode	0	0	0	0	0	1	0	0	1	0	0	1	0	1	0	0	0	0	1	1	0	6
6 Fungi	0	0	0	0	0	0	0	0	1	1	0	0	0	0	0	0	0	0	0	1	0	3
6 Mixed	0	2	0	0	1	3	0	0	0	0	0	0	1	0	1	0	0	2	0	1	0	11
6 Control	0	1	0	0	0	0	0	0	0	0	0	0	0	0	0	1	0	0	0	1	0	3

7 Nematode	0	0	0	0	0	0	0	0	0	1	0	0	0	0	0	0	0	0	0	0	0	1
7 Fungi	0	0	0	0	0	0	1	0	0	0	0	0	0	0	0	0	0	0	1	0	0	2
7 Mixed	0	0	0	0	0	0	0	0	0	0	0	0	0	0	0	1	2	0	0	1	0	4
7 Control	0	0	0	3	1	2	0	0	0	0	0	0	0	0	3	0	1	0	1	4	0	15
8 Nematode	0	0	0	0	0	0	0	0	0	0	0	0	0	0	0	0	0	0	1	0	0	1
8 Fungi	0	0	0	0	0	1	0	0	0	0	0	0	0	0	0	0	0	0	0	0	0	1
8 Mixed	0	0	0	0	1	0	0	0	0	0	0	0	0	1	0	0	0	0	0	0	1	3
8 Control	0	0	0	0	0	2	2	0	0	0	0	0	0	0	0	0	0	0	1	0	0	5
9 Nematode	0	0	0	0	3	1	1	0	0	0	0	0	0	4	0	0	1	0	0	2	0	12
9 Fungi	1	0	0	0	0	0	0	0	0	0	0	0	0	0	1	0	1	0	0	3	0	6
9 Mixed	0	0	0	0	1	0	0	0	0	0	0	0	0	0	0	0	0	0	0	2	0	3
9 Control	0	0	0	0	0	0	0	0	0	0	0	0	0	0	0	1	0	0	0	1	0	2
10 Nematode	0	0	0	0	2	0	0	0	0	0	0	0	0	0	0	0	0	0	1	0	0	3
10 Fungi	0	0	0	0	4	1	0	0	0	1	0	0	0	0	0	0	0	0	0	1	0	7
10 Mixed	0	0	0	0	1	0	0	0	1	0	0	0	0	0	0	1	0	0	0	0	0	3
10 Control	0	0	0	0	1	1	0	1	0	0	0	0	0	0	0	3	0	0	0	0	0	6
